# Aquaporin 3 promotes the stem-like properties of gastric cancer cells via Wnt/GSK-3β/β-catenin pathway

**DOI:** 10.18632/oncotarget.7664

**Published:** 2016-02-24

**Authors:** Yangchun Zhou, Yao Wang, Jianfei Wen, Haijian Zhao, Xuqiang Dong, Zhihong Zhang, Shoulin Wang, Lizong Shen

**Affiliations:** ^1^ Division of Gastrointestinal Surgery, Department of General Surgery, First Affiliated Hospital, Nanjing Medical University, Nanjing, Jiangsu, China; ^2^ Department of General Surgery, Sir Run Run Shaw Hospital, Third Affiliated Hospital, Nanjing Medical University, Nanjing, Jiangsu, China; ^3^ Division of Gastrointestinal Surgery, Department of General Surgery, Affiliated Huai'an Hospital, Xuzhou Medical College, Huai'an, Jiangsu, China; ^4^ Department of Pathology, First Affiliated Hospital, Nanjing Medical University, Nanjing, Jiangsu, China; ^5^ School of Public Health, Nanjing Medical University, Nanjing, Jiangsu, China

**Keywords:** gastric cancer, aquaporin 3, cancer stem cells (CSCs), CD44

## Abstract

Cancer stem cells (CSCs) are believed to contribute to the tumor growth in gastric carcinoma (GC), a common lethal malignancy. This study investigated the effect of aquaporin 3 (AQP3) on stem-like properties of human GC cells. Elevated AQP3 expression was associated with CD44 expression in human GC specimens. Expression of AQP3 and that of CD44 positively correlated with Lauren classification, lymph node metastasis, and lymphovascular invasion. Altering the AQP3 expression had pronounced effects on the tumorigenic potential and self-renewal capacity of the gastric cancer cell lines SGC7901, MGC803, and AGS, both *in vitro* and *in vivo*. Overexpression of AQP3 induced CD44 expression and activation of the β-catenin signaling pathway, whereas silencing AQP3 expression using short hairpin RNA had the opposite effect. Furthermore, pharmacological inhibition of GSK-3β using LiCl impaired the effect of AQP3 knockdown in CSCs, whereas the inhibition of the Wnt/β-catenin pathway by XAV939 blocked the effect of AQP3 overexpression. These results demonstrate that AQP3 promotes stem-like properties of human GC cells by activating the Wnt/GSK-3β/β-catenin signaling pathway.

## INTRODUCTION

Gastric carcinoma (GC) remains as a common lethal malignancy worldwide [1]. In the majority of patients, GC is diagnosed at a later stage, resulting in poor prognosis and therapeutic outcomes [2]. Multistep and multi-factorial processes involving various genetic and molecular alterations, including the activation of various oncogenes and the inactivation of tumor suppressor genes, are involved in the development of GC [3–5]. However, there has been only limited improvement in the treatment of advanced GC.

Mounting evidence suggests that most human cancers, including gastric cancer, are driven by a rare population of cancer cells, the cancer stem cells (CSCs), that display stem cell-like properties [6]. CSCs have the ability to initiate tumor growth and sustain tumor self-renewal. CD44 is an important cell surface marker of breast, pancreatic, colorectal, and prostate cancers [7–10]. Recent studies have identified CD44 as the most significant marker of gastric CSCs [11, 12].

Aquaporins (AQPs) are a family of integral membrane proteins that transport water and, in some cases, water and glycerol (“aquaglyceroporins”) [13, 14]. Our previous study demonstrated that AQP3 is overexpressed in GC tissues and that its expression is associated with histological classification, lymph node metastasis, and lymphovascular invasion [15, 16]. That the upregulation of AQP3 promotes the proliferation and migration of GC cells via promoting the epithelial-mesenchymal transition (EMT) suggested that AQP3 is involved in the carcinogenesis and progression of GC [17].

Earlier studies have shown that *Helicobacter pylori* (*H. pylori*) infection causes the generation of cells with cancer stem cell properties via EMT [12]. Our earlier studies have shown that *H. pylori* infection upregulates AQP3 in GC and that AQP3 promotes EMT in GC [17, 18]. However, whether AQP3 altered the stem-like properties of GC cells remained unknown. In the present study, we evaluated the clinical significance of the expression of AQP3 and CD44 in human GC tissues, and examined their correlation. Additionally, we investigated the effect of AQP3 on CD44 expression and stem-like properties of gastric cancer cells and elucidated the mechanism involved in this effect. Our results demonstrate that AQP3 increases CD44 expression through the Wnt/GSK-3β/β-catenin signaling pathway and promotes the stem-like properties of GC cells.

## RESULTS

### AQP3 expression correlates with CD44 expression in GC tissues

**Figure 1:**
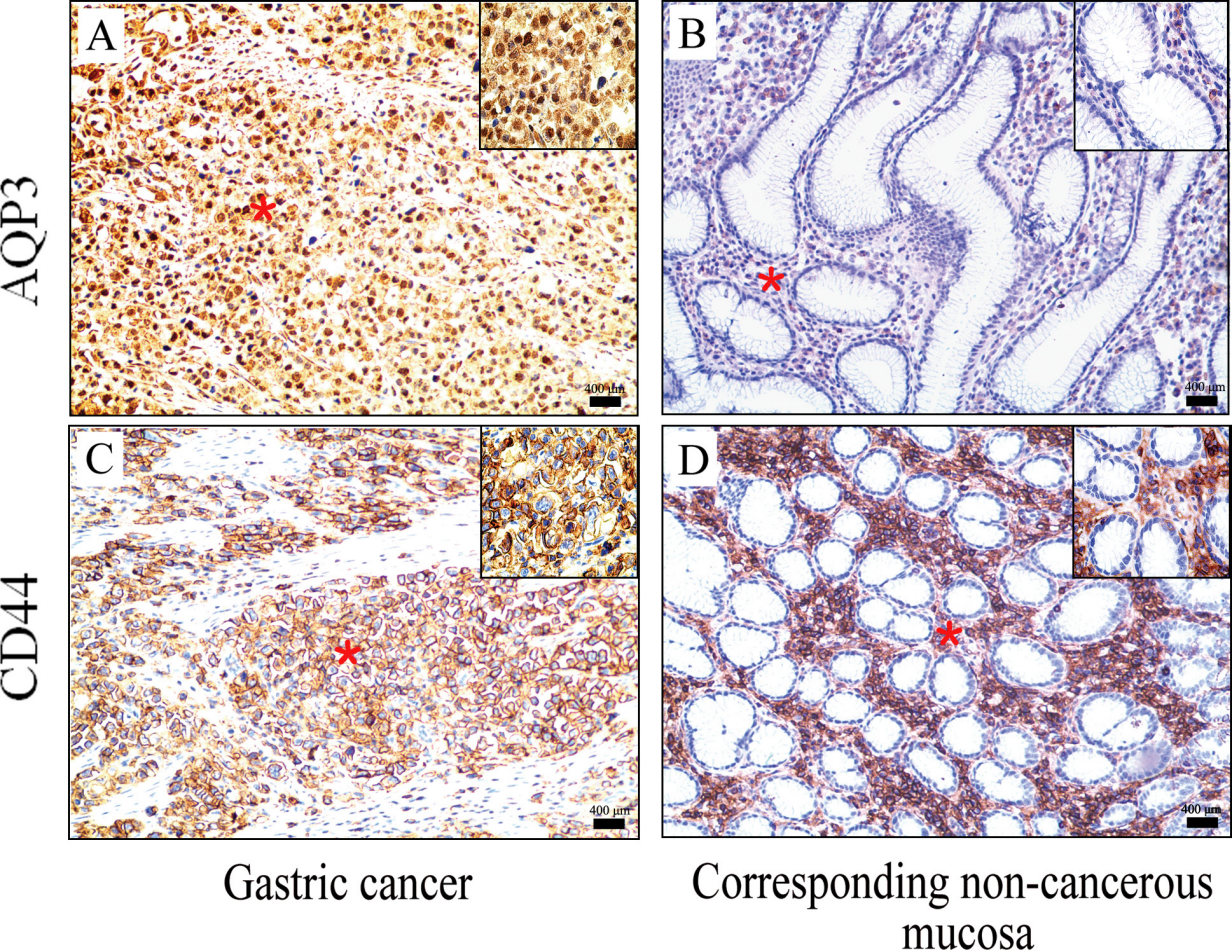
Expression of AQP3 and CD44 in GC tissues and the corresponding non-cancerous mucosal tissues is analyzed by IHC Eighty patients with GC were randomly enrolled in this study, and strong AQP3 and CD44 immunoreactivity was observed in GC tissue compared with the corresponding non-cancerous mucosal tissue. The original magnification of each big Figure was × 100. The right upper quarter indicated the magnified photomicrograph of the corresponding portion (red star) in each figure, and its original magnification was × 400.

As shown in Figure 1 and Table 1, the GC tissues expressed significantly higher levels of AQP3 and CD44 compared to the corresponding non-cancerous mucosa. This result was consistent with the results of our previous study [15–17] and that of others [19].

**Table 1:** Correlations of AQP3 expression or CD44 expression in GC tissues and corresponding non-cancerous mucosa tissues

Proteins	Gastric cancer tissues	Gastric non-cancerous mucosa tissues	χ2	*P*-value
AQP3			50.6333	< 0.001
Positive	63	18		
Negative	17	62		
CD44			58.284	< 0.001
Positive	48	3		
Negative	32	77		

The correlation of AQP3 and CD44 expression levels with the clinicopathological features of GC in patients was evaluated (Table 2). The results showed that elevated expression of AQP3 in cancer tissues was associated with the Lauren classification (*P =* 0.034), lymph node metastasis (*P =* 0.006), and lymphovascular invasion (*P =* 0.024). CD44 expression also correlated significantly with Lauren classification (*P =* 0.002), lymph node metastasis (*P =* 0.049), and lymphovascular invasion (*P =* 0.044). Furthermore, AQP3 expression positively correlated with the expression of CD44 in GC tissues (*P =* 0.019, Table 3). Together, these results indicated that AQP3 might be involved in the induction of gastric CSCs.

**Table 2:** Correlation between AQP3, CD44 expression and clinicopathological features in GC

Clinicopathological features	*n*	AQP3			CD44		
		+	–	*P*-value	+	–	*P*-value
Age				0.817			0.194
< 60	33	24	7		17	16	
≥ 60	47	39	10		31	16	
Sex				0.305			0.261
Male	58	44	14		37	21	
Female	22	19	3		11	11	
Lauren classification				0.034			0.002
Intestinal	48	34	14		22	26	
Diffuse and mixed	32	29	3		26	6	
Tumor size				0.389			0.242
≥ 3	54	44	10		30	24	
< 3	26	19	7		18	8	
Tumor location				0.604			0.818
Upper third	28	21	7		17	11	
Middle third	20	15	5		13	7	
lower third	32	27	5		18	14	
Depth of tumor invasion				0.879			0.923
Localized in subserosa	27	21	6		16	11	
Beyond subserosa	53	42	11		32	21	
Lymph node metastasis				0.006			0.049
N0	25	15	10		11	14	
N1- N3	55	48	7		37	18	
pTNM stage				0.584			0.404
I/II	33	25	8		18	15	
III/IV	47	38	9		30	17	
Lymphovascular invasion				0.024			0.044
Absence	52	37	15		27	25	
Presence	28	26	2		21	7	

**Table 3:** Correlation between expression levels of AQP3 and CD44 in GC tissues by IHC

	AQP3 (+)	AQP3 (−)	χ^2^	*P*-value
CD44 (+)	42	6	5.490	0.019
CD44 (−)	21	11		

### AQP3 promotes the ability of GC cells to form spheroids

**Figure 2:**
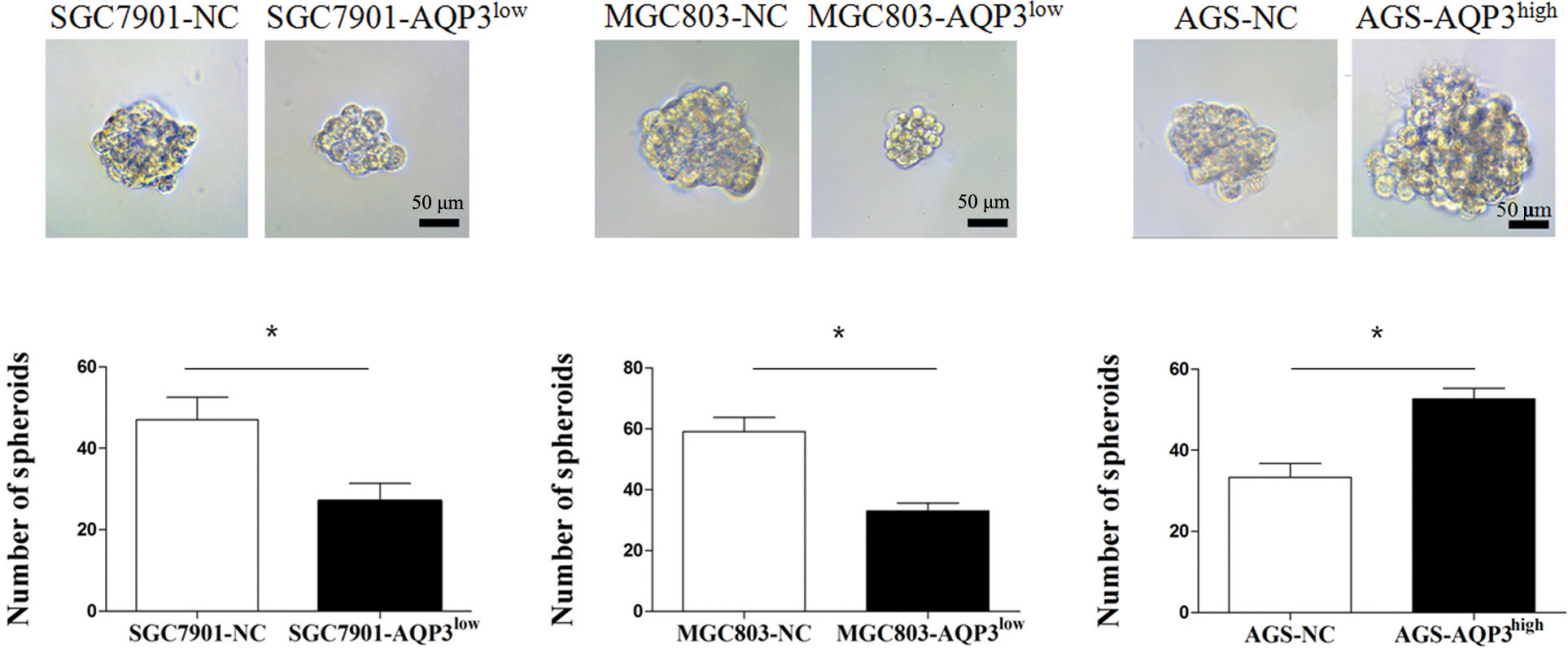
AQP3 promotes the ability of GC cells to form spheroids Spheroids were significantly lower in the cultures of SGC7901-AQP3^low^ and MGC803-AQP3^low^ cells and higher in that of AGS-AQP3^high^ cells. Data are expressed as the mean ± SE of the results from three independent experiments. *P < 0.05 compared with the null control (NC).

The spheroid formation assay was performed to assess the effect of AQP3 on the self-renewal capacity of the GC cells. As shown in Figure 2, the number of spheroids formed decreased significantly when AQP3 expression in SGC7901 and MGC803 cells was downregulated using shRNA (*P* < 0.05). In contrast, AQP3 upregulation in AGS cells promoted the formation of spheroids (*P* < 0.05), indicating that AQP3 might act to promote the self-renewal of GC cells.

### AQP3 increases the tumorigenic capacity of GC cells *in vitro* and *in vivo*

**Figure 3:**
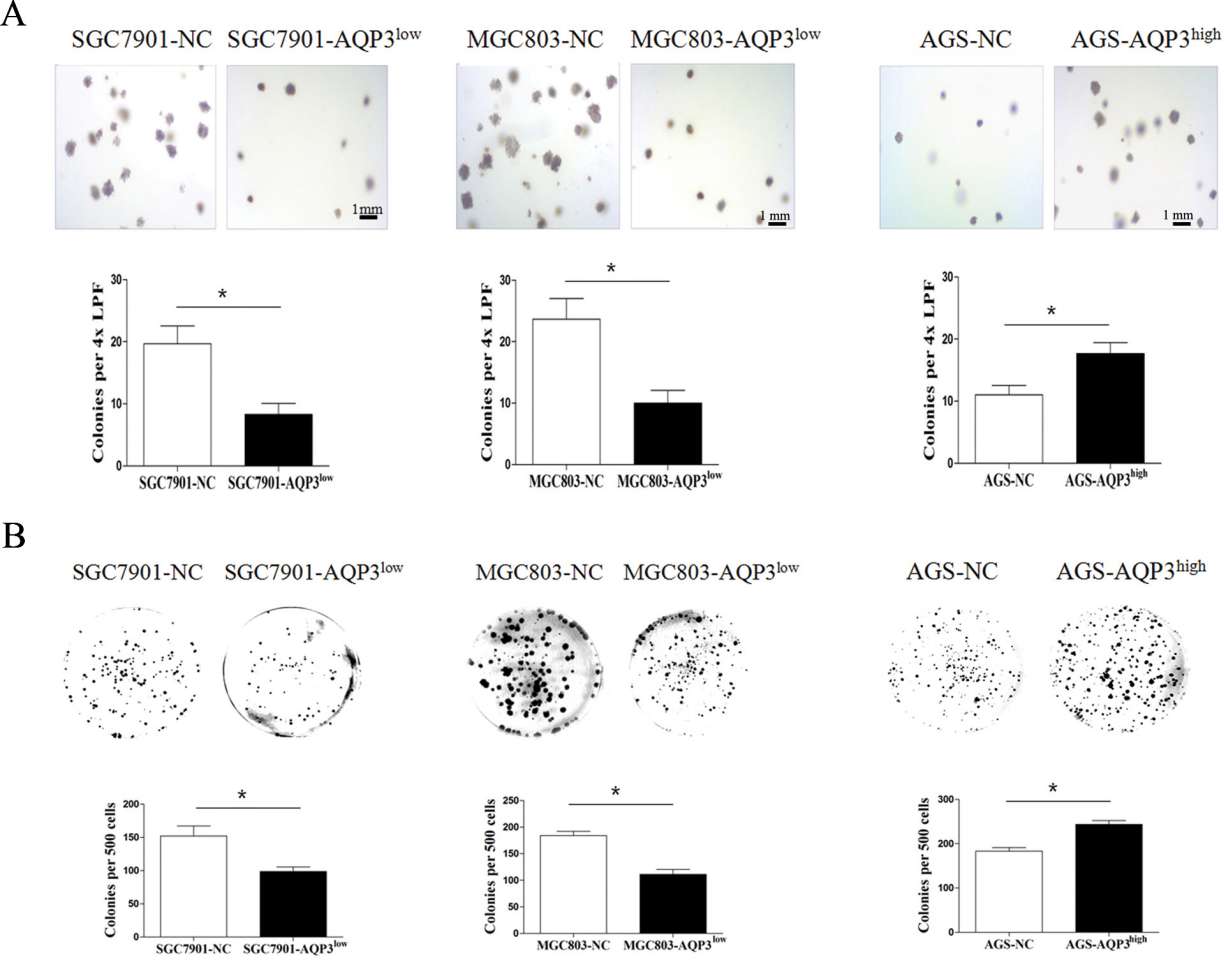
AQP3 promotes the tumorigenic potential of GC cells *in vitro* (**A**) Colonies were significantly lower in agar cultures of SGC7901-AQP3^low^ and MGC803-AQP3^low^ cells and higher in the cultures of AGS-AQP3^high^ cells. (**B**) Colonies in the plate colony formation assay were also lower in the cultures of SGC7901-AQP3^low^ and MGC803-AQP3^low^ cells and higher in cultures of AGS-AQP3^high^ cells. Data are expressed as the mean ± SE from three independent experiments. *P < 0.05 compared with the null control (NC).

**Figure 4:**
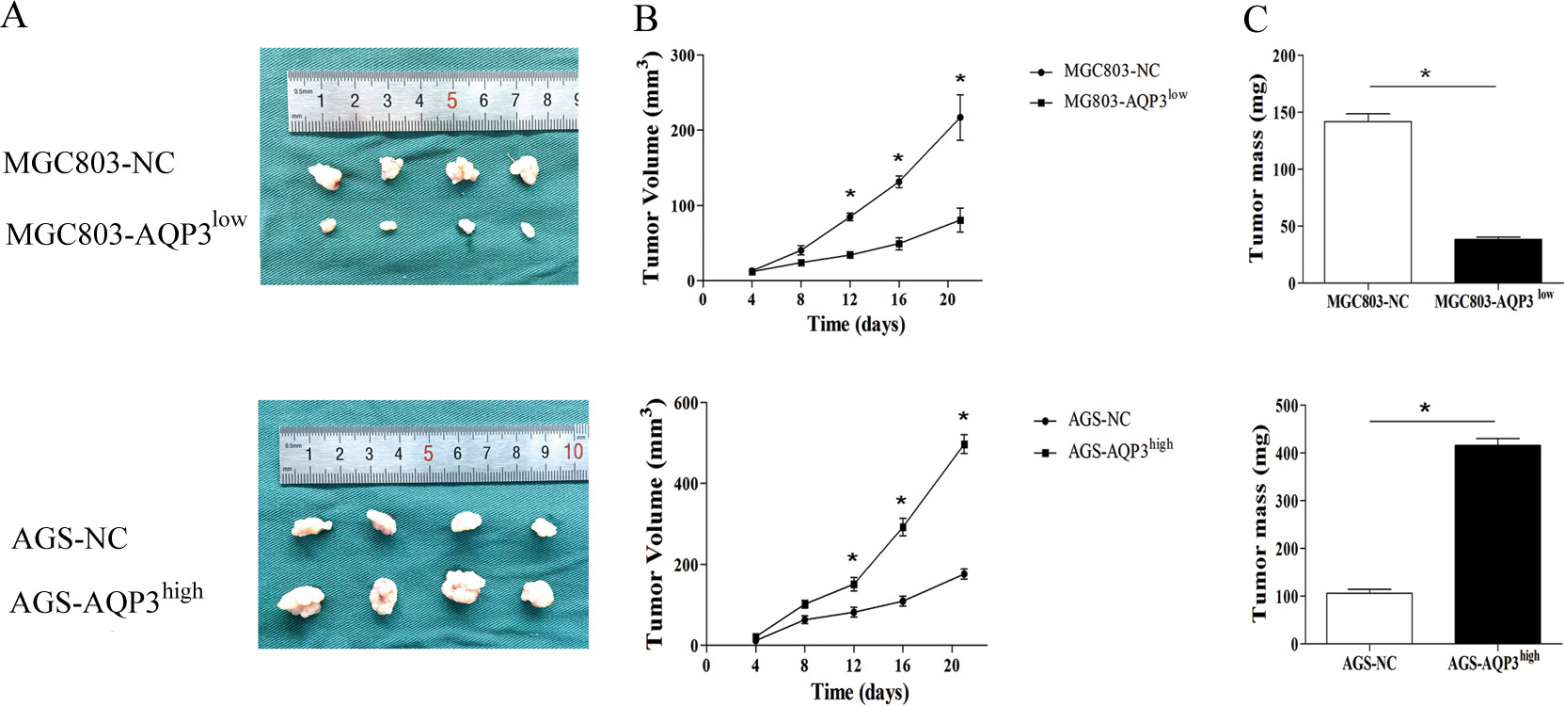
AQP3 promotes tumorigenic potential of GC cells *in vivo* (**A**) MGC803-NC, MGC803-AQP3^low^, AGS-NC, and AGS- AQP3^high^ stable cells were injected subcutaneously into nude mice. (**B**) Tumor volume was measured every 4 days. (**C**) After 3 weeks, the mice were sacrificed and the tumors in individual mice were weighed. Each group consisted of four mice. Data are expressed as the mean ± SE. *P < 0.05 compared with the null control (NC).

To assess the effect of AQP3 on the tumorigenic capacity of GC cells, we evaluated the anchorage-independent colony-forming ability of SGC7901, MGC803, and AGS cells. The colony-forming ability of SGC7901 and MGC803 cells drastically diminished upon the suppression of AQP3 expression. Further, the colony-forming ability of AGS cells improved following the overexpression of AQP3 (Figure 3A). The clonogenic potential of the GC cells was also assessed by the plate colony formation assay (Figure 3B). Next, using a nude mouse xenograft model, we investigated whether AQP3 promoted tumorigenesis *in vivo*. The results showed that the mice transplanted with MGC803or AGS cells developed solid tumors within 3 weeks of transplantation. Compared with the null control group, the tumor volume and weight were lower in the group that received AQP3 knockdown MGC803 cells (*P* < 0.05), whereas that in the group that received AQP3-overexpressing AGS cells was higher (*P* < 0.05) (Figure 4). These findings suggested that AQP3 expression significantly improved the tumorigenic potential of GC cells.

### AQP3 increases the expression of CD44 in GC cells

**Figure 5:**
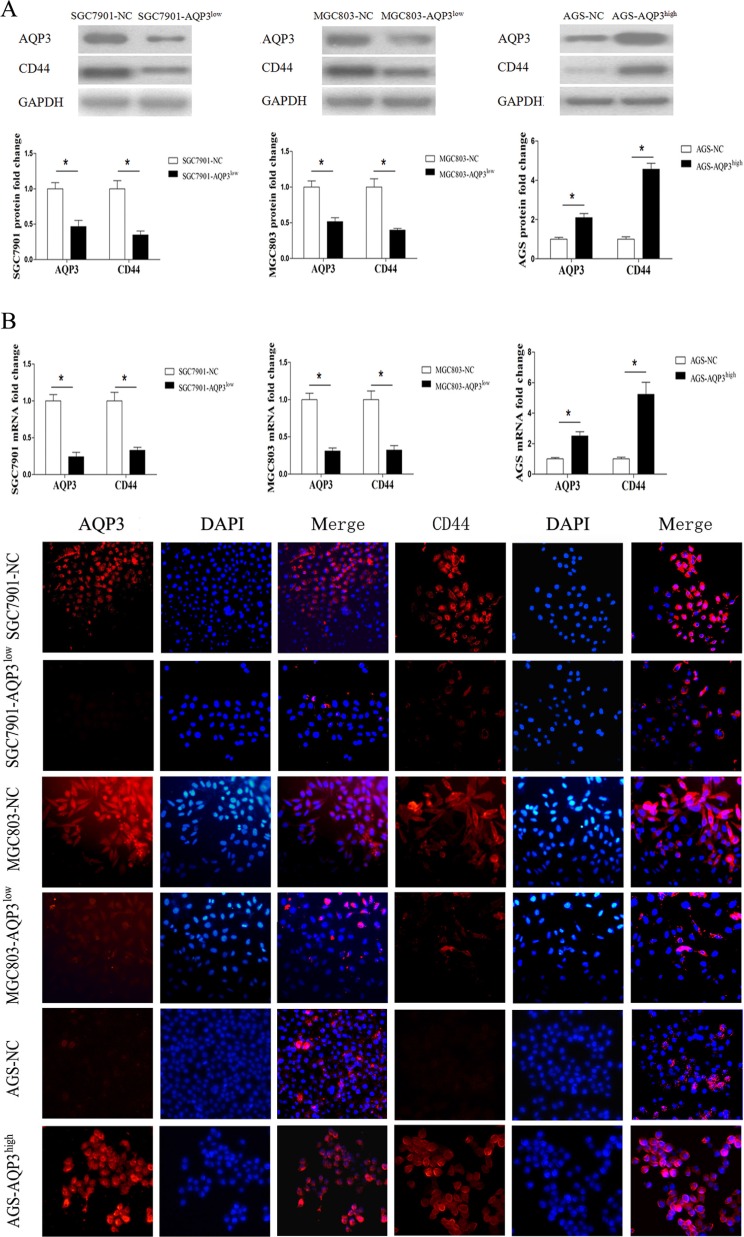
AQP3 increases the expression of CD44 in GC cells (**A**) Expression levels of AQP3 and CD44 in SGC7901, MGC803, and AGS cells were determined by Western blot method. GAPDH was used as internal control. The relative expression levels of proteins in different groups were compared with those in the null control (NC). (**B**) The mRNA expression levels of AQP3 and CD44 were quantified by RT-qPCR analysis. Data are expressed as the mean ± SE of results from three independent experiments. *P < 0.05 compared with the null control (NC). (**C**) Immunofluorescence assays for the detection of AQP3 and C44. The target proteins were detected using the respective antibodies (red), and nuclei were stained with DAPI (blue). Original magnification, × 200.

**Figure 6:**
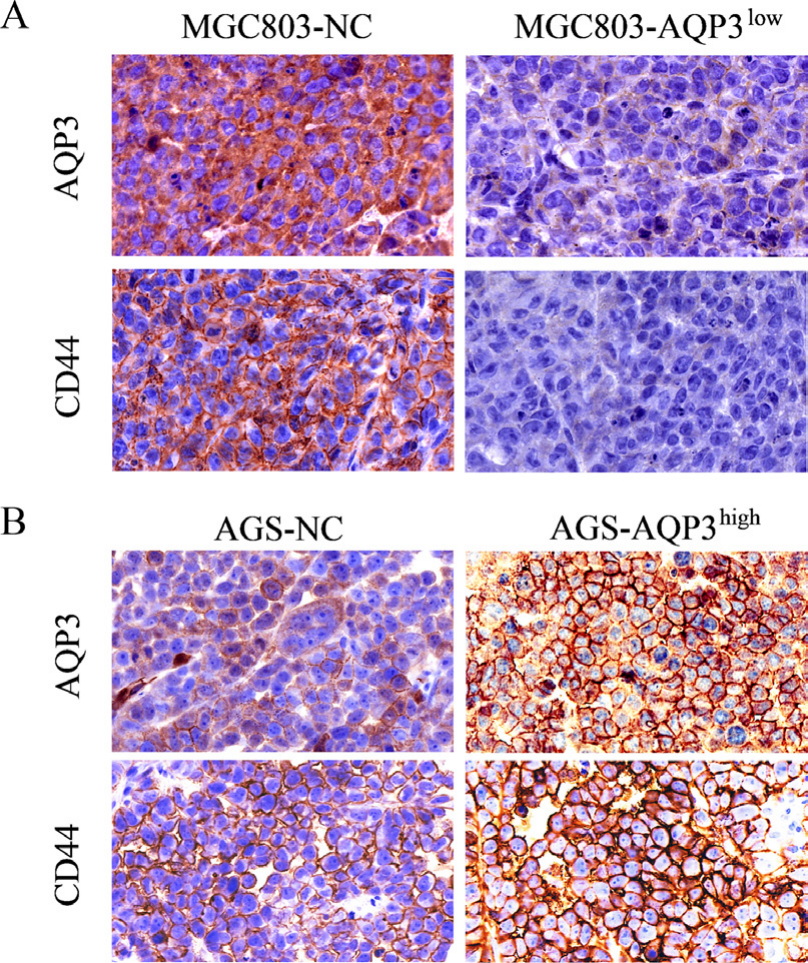
Expression of AQP3 and CD44 proteins is determined in tumors of mice by IHC Poor AQP3 and CD44 immunoreactivity in tumors of mice were identified in MGC803-AQP3^low^ group compared with MGC803-NC group. Strong AQP3 and CD44 immunoreactivity in tumors of mice were identified in AGS-AQP3^high^ group compared with AGS-NC group. Original magnification, × 400.

CD44 is the primary marker of gastric CSCs. Therefore, we studied whether AQP3 promoted the stem-like properties of GC cells by augmenting CD44 expression. As shown in Figure 5A, AQP3 knockdown abrogated CD44 expression in SGC7901 and MGC803 cells, whereas its overexpression upregulated CD44 expression in AGS cells. The results of RT-qPCR analysis (Figure 5B) and immunofluorescence staining (Figure 5C) further confirmed these results. We also performed immunohistochemical analysis of AQP3 and CD44 expression in mouse tumors and found that tumors in the MGC803-AQP3^low^ group expressed low levels of AQP3 and CD44, whereas that in the AGS-AQP3^high^ group expressed high levels of AQP3 and CD44 (Figure 6). Taken together, these findings suggested that AQP3 promoted GC cell self-renewal via CD44.

### AQP3 activates β-catenin signaling to increase CD44 expression

**Figure 7:**
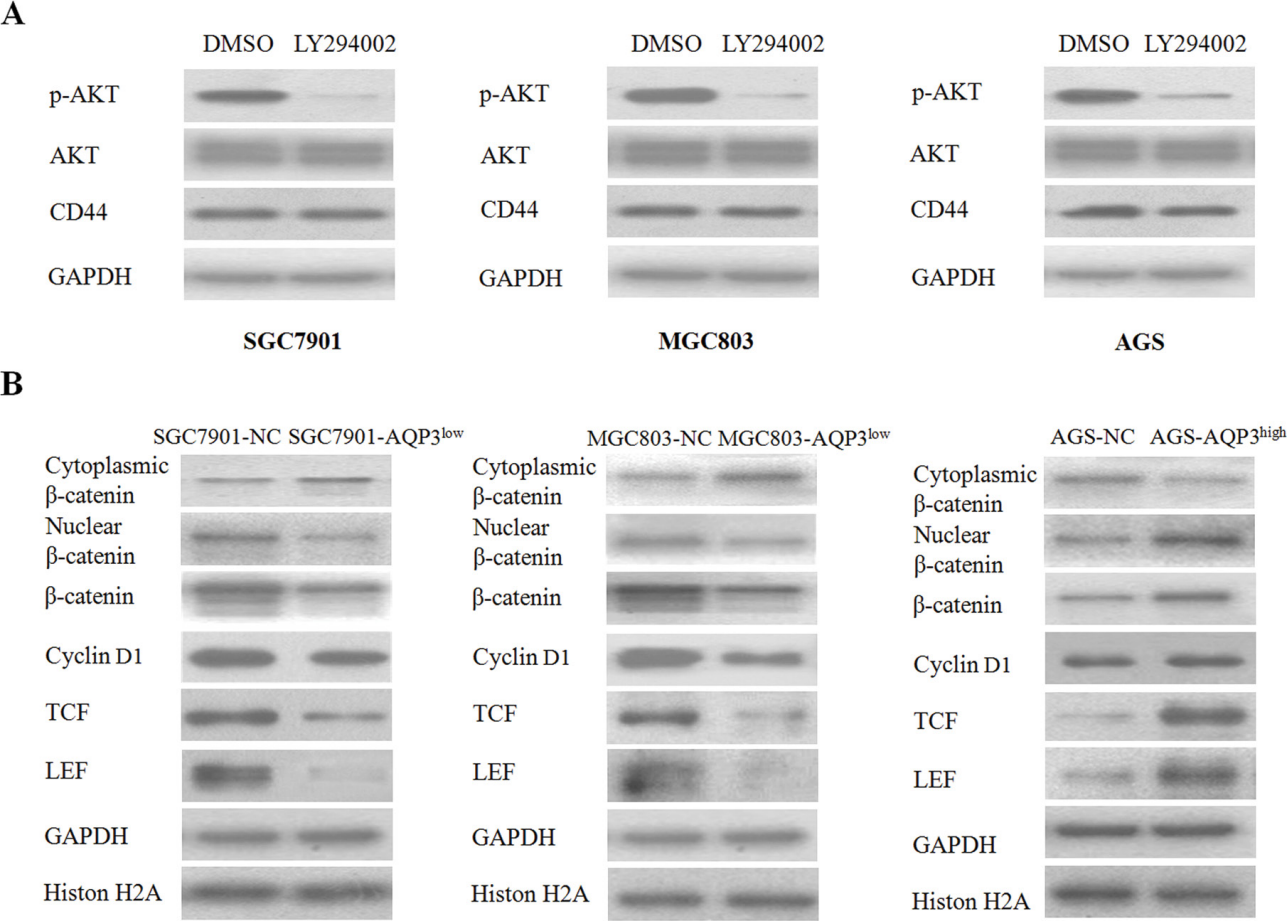
AQP3 activates β-catenin signaling to increase CD44 expression (**A**) SGC7901, MGC803, and AGS cells were treated with the PI3K/AKT inhibitor, LY294002. (**B**) The β-catenin signaling-related proteins were analyzed by Western blot method. GAPDH was used as internal control. The relative expression levels of proteins were compared with the null control (NC).

Our previous studies demonstrated that in GC, AQP3 promotes EMT via the PI3K/AKT pathway [17]. However, whether AQP3 increases CD44 through this pathway remained unknown. Therefore, we treated SGC7901, MGC803, and AGS cells with LY294002, a specific PI3K/AKT inhibitor. Although phospho-AKT (Ser473) expression was very low in the treated cells, a marked change in the CD44 expression was not observed (Figure 7A). Earlier studies have reported that CD44 is a downstream target of the β-catenin signaling pathway [20, 21]. Therefore, we examined whether AQP3 increased CD44 via this pathway. As shown in Figure 7B, the expression of β-catenin in the nucleus increased significantly following AQP3 overexpression in AGS cells and decreased following AQP3 knockdown in SGC7901 and MGC803 cells. The expressions of total β-catenin, LEF, TCF, and Cyclin D1 were also upregulated by AQP3 overexpression. These results indicated that AQP3 increased CD44 expression in GC cells via a mechanism involving β-catenin activation.

**Figure 8:**
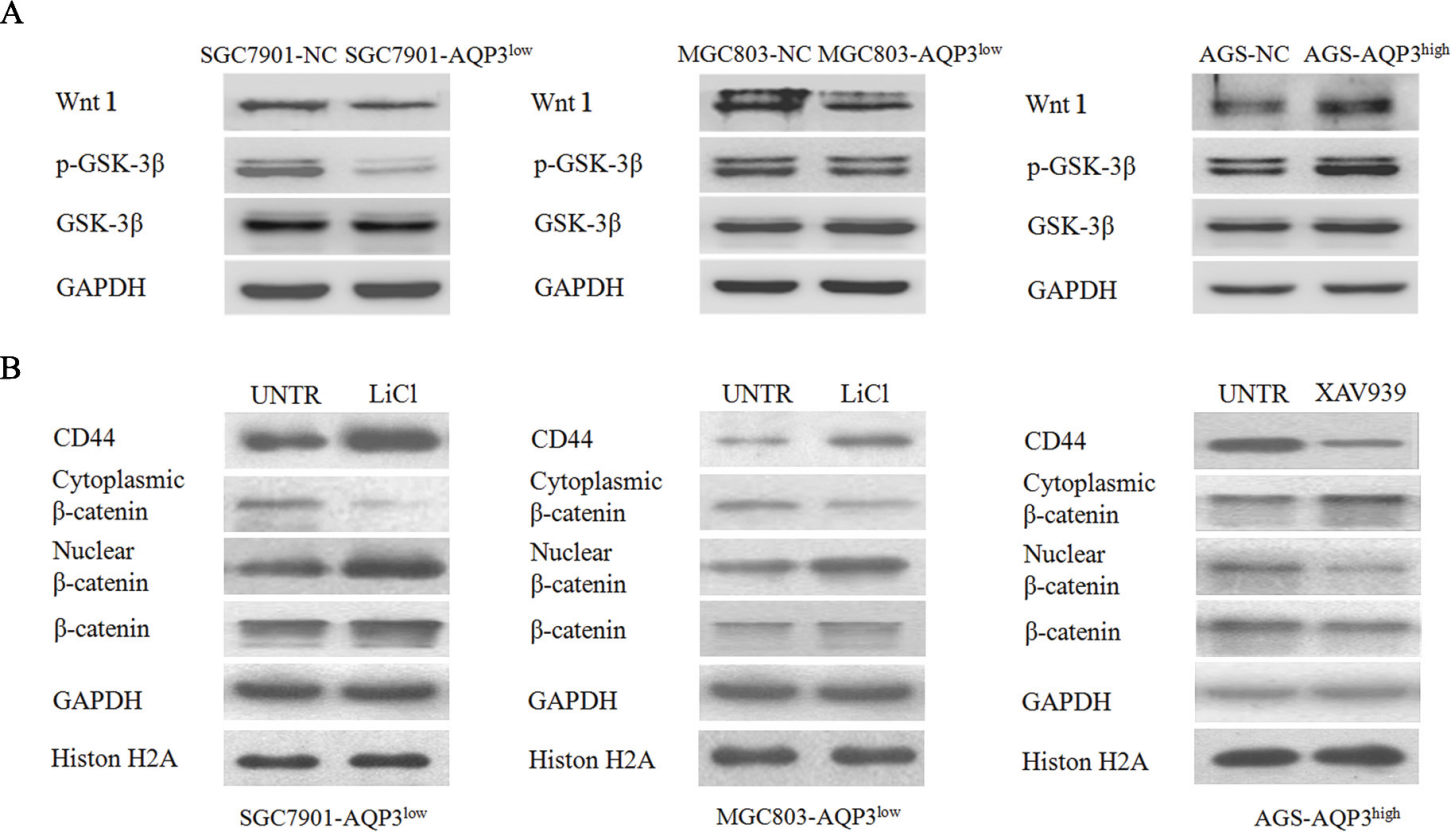
AQP3 activates β-catenin signaling through Wnt and GSK-3β (**A**) Expression levels of Wnt and GSK-3β in SGC7901, MGC803, and AGS cells were determined using Western blot method. (**B**) SGC7901-AQP3^low^ and MGC803-AQP3^low^ cells were treated with LiCl, and AGS-AQP3^high^ cells were treated with XAV939. GAPDH was used as internal control. The relative expression levels of proteins were compared with the null control (NC).

**Figure 9:**
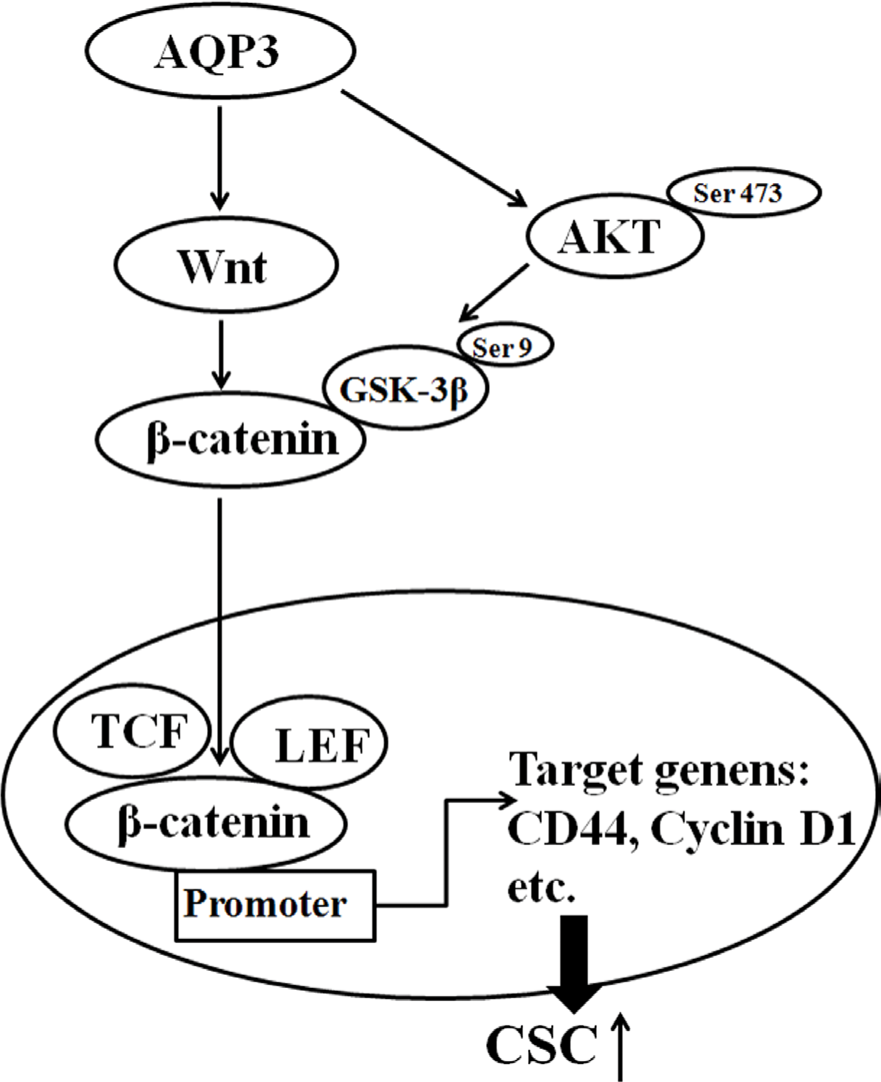
The suggested pathway for AQP3 in inducing the stem-like properties of GC cells

Therefore, we investigated the mechanism by which AQP3 activated β-catenin. As shown in Figure 8A, the expression of Wnt and phospho-GSK-3β (Ser9) increased following AQP3 overexpression in AGS cells, whereas their expression decreased following the knockdown of AQP3 in SGC7901 and MGC803 cells. Two specific inhibitors were used to study the potential roles of Wnt and GSK-3β in AQP3-mediated regulation of β-catenin. XAV939, an inhibitor of Wnt pathway, inhibited the nuclear translocation of β-catenin and CD44expression in AGS cells overexpressing AQP3. However, LiCl, a GSK-3β inhibitor, rescued the expression of nuclear β-catenin and CD44 in AQP3-knockdown SGC7901 and MGC803cells (Figure 8B). These results demonstrated that AQP3 increased CD44 expression through Wnt/GSK-3β/β-catenin signaling pathway and promoted the stem cell-like properties of GC cells (Figure 9).

## DISCUSSION

Recent studies suggest that tumor initiation, progression, and metastasis in many human cancers originate from a specific subpopulation of cancer cells with stem-like properties [22]. CSCs are those cells that possess high tumor-initiating capacity with the ability to self-renew and differentiate into a limited number of cell types [6]. Human gastric CSCs with these characteristics have been identified by various groups using specific cell surface markers and *in vitro* as well as *in vivo* assays [11, 12, 23]. CD44 is the most significant marker of CSCs [11, 24]. Our previous studies revealed that AQP3 overexpression is involved in the tumorigenesis and progression of GC [15–17, 25] and that AQP3 upregulation promotes EMT in GC cells [17]. Pioneering studies have demonstrated the involvement of EMT in the generation of CSCs [26–28]. EMT induction in cancer cells results in the acquisition of invasiveness and metastatic properties. The metastatic outgrowth is also thought to be associated with the ability to of the cells to self-renew, a defining trait of CSCs [28, 29], suggesting that CSCs are produced partly through EMT. Although our earlier studies showed that AQP3 promotes EMT, whether this protein promoted the stem-like properties of cancer cells in GC remained unknown.

In the present study, we found that AQP3 is overexpressed in GC tissues and that its overexpression is associated with Lauren classification, lymph node metastasis, and lymphovascular invasion. These results are consistent with our previous findings [15–17]. Our results showed that CD44 is also upregulated in GC tissues, which was also associated with Lauren classification, lymph node metastasis, and lymphovascular invasion. To our knowledge, this is the first study to identify the positive correlation between AQP3 overexpression and CD44 expression in GC tissues. Our results strongly suggest that AQP3 expression is associated with the induction of CSCs in human GC tissues.

The self-renewal capacity and the tumorigenic potential are the major traits of CSCs [30]. Several techniques allow the study of these properties. These techniques include the mice xenograft models for studying the tumorigenesis *in vivo* from transplanted cells, and the *in vitro* spheroid formation and migration assays. The widely used spheroid formation assay relies on the *in vitro* formation of spheroids under non-adherent culture conditions in defined media after several days of culture. This assay is ideal for identifying CSCs, as it depends on the capacity of cells to self-renew and form three-dimensional spheres similar to a tumor. The results of the present study showed that AQP3 promoted the ability of GC cells to form spheroids and significantly increased their clonogenic potential both *in vitro* and *in vivo*. These results clearly suggest that AQP3 promotes the stem-like properties of GC cells. Our results also showed that AQP3 overexpression is associated with the expression of CD44, a known gastric CSCs marker [24, 31], in GC tissues. These results clearly suggested that AQP3 promotes stem-like properties of GC cells by augmenting CD44 expression. The results of Western blotting, RT-qPCR analysis, and immunofluorescence staining demonstrated that AQP3 positively increases the expression of CD44 in GC cells. Together, these results indicated that AQP3 promotes GC cell self-renewal via a mechanism involving CD44.

We next sought to identify the signaling mechanism by which AQP3 increased CD44 expression in GC cells. Our previous study had shown that AQP3 promotes EMT in GC cells through PI3K/AKT pathway. However, in the present study, we found that LY294002, a specific PI3K/AKT inhibitor, had no effect on the expression of CD44 in GC cells. This result suggested that the PI3K/AKT pathway was not involved in the regulation of CD44 expression by AQP3 in GC cells. Interestingly, we found that the expression of β-catenin in the nucleus significantly increased following AQP3 overexpression and decreased after AQP3 knockdown. Additionally, AQP3 upregulated the expression of LEF and TCF, which form a complex with β-catenin in the nucleus. These results indicated that AQP3might have acted to increase the activity of β-catenin in the nucleus and promote the expression of CD44.

The phosphorylation of β-catenin by GSK-3β leads to its degradation via the ubiquitin/proteasome pathway. Our results showed that the increase in β-catenin levels observed following AQP3 overexpression was associated with an increase in phospho-GSK-3β levels, which inactivated GSK-3β. We also found that the treatment with LiCl, a GSK-3β-specific inhibitor, reversed the effect of AQP3 downregulation on phospho-GSK-3β, β-catenin, and CD44 levels in GC cells. These data clearly suggested that the inactivation of GSK-3β by AQP3 is a possible mechanism that increased nuclear β-catenin levels. A specific Wnt pathway inhibitor (XAV939) attenuated the AQP3-induced activation of β-catenin and CD44 expression in GC cells overexpressing AQP3. Together, the present results demonstrate that the Wnt/GSK-3β/β-catenin signaling pathway is involved in the promotion of stem-like properties by AQP3 in human GC cells (Figure 9).

In summary, for the first time, we demonstrated that AQP3 increases CD44 expression through Wnt/GSK-3β/β-catenin signaling pathway and promotes the stem-like properties of cancer cells in GC. The present results point toward the key role of AQP3 in the tumorigenesis and progression of gastric carcinoma, and provide promising new avenues for the development of therapeutic strategies for treating gastric cancer.

## MATERIALS AND METHODS

### Human gastric tissue specimens

Gastric adenocarcinoma patients (*n =* 80; median age, 59 years; range, 36–76 years) were randomly enrolled between January 2012 and December 2013 at the Department of General Surgery of the First Affiliated Hospital of Nanjing Medical University. All patients were diagnosed pathologically according to the American Joint Committee on Cancer (AJCC) criteria. No patient had received chemotherapy or radiotherapy before surgery. Tumor samples and the corresponding non-cancerous mucosal tissue were collected from all patients immediately after resection and were snap frozen in liquid nitrogen. The correlation between the clinicopathological characteristics of the patients and the expression of AQP3 and CD44 was analyzed individually. These characteristics are listed in Table 2. Cases with distant metastasis were not presented in this study. Patients provided their written informed consent. Samples were stored in the hospital database for studies. This study was approved by the Nanjing Medical University Institutional Review Board, and complied with the Helsinki Declaration.

### Immunohistochemical detection of AQP3 and CD44 in tissues

Expression of AQP3 and CD44 in specimens was analyzed by immunohistochemistry (IHC) as described previously [16, 18]. Polyclonal rabbit anti-AQP3 antibody was obtained from Santa Cruz Biotechnology (Santa Cruz, CA) and the monoclonal antibody against CD44 was purchased from Cell Signaling Technology (Beverly, MA). The percentage of immune-positive tumor cells (scale 0–100%) falling within a staining intensity ranging from 0 to 3+ was scored by two pathologists. When an antibody stained more than 25% of the cells at intensities ranging from 2 to 3+, the corresponding antigen was considered to be expressed in the tumor cells.

### Cell culture

The human GC cell lines SGC7901, MGC803, and AGS (CBTCCCAS, Shanghai, China) were cultured in RPMI-1640 (Life Technologies, Gibco BRL, Grand Island, NY, USA) supplemented with 10% fetal bovine serum (FBS; Invitrogen, Carlsbad, CA, USA), penicillin/streptomycin (1:100 dilution; Sigma, St. Louis, MO, USA), and 4 mM glutamine (Life Technologies, Gibco BRL) at 37°C in a humidified atmosphere of 5% CO_2_.

### Lentiviral transduction

Lentiviruses carrying AQP3 short hairpin RNA (shRNA), AQP3, or the corresponding empty GFP were constructed by GeneChem Biomedical Co. Ltd (Shanghai, China). The transduction was performed according to the manufacturer's recommended protocol. Stable cell lines were established by selecting the transduced cells with 2 μg/mL puromycin (Sigma-Aldrich, St. Louis, MO) for one week. The expression of AQP3 was analyzed by real-time quantitative polymerase chain reaction (RT-qPCR) and Western blot methods.

### Soft agar colony formation assay

The anchorage-independent cell proliferation was assessed by the soft agar assay. Typically, 3 × 10^3^ cells were mixed with 0.35% agar solution in RPMI-1640 containing 10% FBS and layered on top of a 0.5% base agar layer in 6-well plates. The plates were incubated for 2 weeks at 37°C in an atmosphere of 5% CO_2_. Cultures were fed every 3 days with fresh RPMI-1640 medium supplemented with 10% FBS. After 14 days of continuous culture, colonies containing more than 30 cells were counted under a microscope.

### Plate colony formation assay

The plate colony formation assay was carried out to assess the clonogenic potential of the cells. Briefly, 500 single viable cells in RPMI-1640 containing 10% FBS were plated in 6-well plates. The cells were incubated at 37°C in an atmosphere of 5% CO_2_ for 14 days. Following this, the colonies formed were stained with Crystal Violet Solution (Beyotime Institute of Biotechnology, Henan, China), washed with water, and counted.

### Spheroid formation assay

Spheroid formation assay was performed according to Hwang's reported method [30]. Briefly, the cells were suspended in stem cell medium composed of RPMI-1640 supplemented with 2% B27 supplement (Invitrogen), human recombinant epidermal growth factor (rhEGF, 20 ng/mL) (PeproTech, Rocky Hill, NJ, USA), and basic fibroblast growth factor (bFGF, 20 ng/mL) (PeproTech, Rocky Hill, NJ, USA) in ultra-low attachment 96-well plates (Corning Life Science, Acton, MA, USA) for 14 days. The number of spheroids formed per well was estimated by counting under a light microscope.

### Real-time quantitative polymerase chain reaction (RT-qPCR)

The RT-qPCR analysis was performed according to our reported method [16, 18]. GAPDH served as reference, and the observed gene expression level was normalized to the level of GAPDH. The following primer pairs were used in RT-qPCR experiments: 5′-CTCGTGAGCCCTGGATCAAGC-3′ (sense) and 5′-AAAGCTGGTTGTCGGCGAAGT-3′ (antisense) for AQP3, 5′-CCAGATGGAGAAAGCTCTGA-3′ (sense) and 5′-GTCATACTGGGAGGTGTTGG-3′ (antisense) for CD44, 5′-CGCTGAGTACGTCGTGGAGTC-3′ (sense) and 5′-GCTGATGATCTTGAGGCTGTTGTC-3′ (antisense) for GAPDH. All RT-qPCR reactions were performed in triplicate.

### Western blot assay

Western blot assay was performed to analyze the expression of proteins according to our reported methods [16, 18]. The nuclear and cytoplasmic fractions were prepared using a Nuclear and Cytoplasmic Protein Extraction Kit (Beyotime Institute of Biotechnology, Henan, China). The following antibodies were used for this analysis: antibodies against AQP3 and Wnt1 (Santa Cruz Biotechnology, Santa Cruz, CA), and specific antibodies against CD44, AKT, phospho-AKT (Ser473), GSK-3β, phospho-GSK-3β (Ser9), β-catenin, LEF, Cyclin D, TCF (Cell Signaling Technology, Beverly, MA), Glyceraldehyde-3-phosphatedehydrogenase (GAPDH), and Histone H2A antibodies (Beyotime Institute of Biotechnology, Henan, China). Protein expression was quantified by densitometric analysis, and the expression levels were normalization against that of GAPDH. LY294002, LiCl and XAV939 were obtained from Sigma Aldrich (St. Louis, MO).

### Immunofluorescence staining

Immunofluorescence staining was performed according to Huang's reported method [32]. The primary antibodies used for western blot assay were used for this purpose. The secondary antibodies were obtained from Beyotime (Beyotime Institute of Biotechnology, Henan, China). Stained samples were imaged using a fluorescence microscope (Olympus Corporation, Tokyo, Japan).

### Tumorigenicity *in vivo*

All animal experiments were conducted according to the guidelines of the Nanjing Medical University Institutional Animal Care and Use Committee. A total of 16 four-week-old nude mice (BALB/c nude mice, Vitalriver, China) were randomly divided into four groups. For the analysis of tumorigenicity, 5 × 10^4^ cells from MGC803-NC, MGC803-AQP3^low^, AGS-NC, or AGS-AQP3^high^ stable cell lines were injected subcutaneously into the flanks of the mice. Tumor volume was measured every 4 days and was calculated based on the following modified ellipsoidal formula: tumor volume = (length × width^2^) × 0.5 [32, 33]. The mice were euthanized after 3 weeks. Thereafter, the tumor tissues were removed, weighed, fixed with 4% formalin, and embedded in paraffin. Immunohistochemical analysis was performed as described elsewhere [16, 18] using antibodies against AQP3 (Santa Cruz, CA) and CD44 (Cell Signaling Technology, Beverly, MA).

### Statistical analysis

Data were expressed as the mean ± SE. In the experiments involving protein expression, the values were representative of three independent experiments. Pearson's Chi-square test was used to examine the association between protein expression levels and various clinicopathological parameters. Statistical analysis of the quantitative data between the control and the treatment groups was performed by analysis of variance. These analyses were performed using the SPSS (version 19.0, SPSS Inc., Chicago, IL, USA) software, and *P* < 0.05 was considered statistically significant.

## Abbreviations

GC: Gastric carcinoma
AQP: Aquaporin
CSCs: Cancer stem cells
EMT: epithelial-mesenchymal transition
shRNA: short hairpin RNA.
